# Supplementary material for: EnzML: multi-label prediction of enzyme classes using InterPro signatures
Source: BMC Bioinformatics. 2012 Apr 25;13:61. doi: 10.1186/1471-2105-13-61 (PMC3483700; doi:10.1186/1471-2105-13-61)
Supplement: Addtional file 5 — The Java code to format the data files, evaluate and predict. The file enzml_java_code.tar.gz contains the Java code used to format database data to ARFF and XML formats, to execute cross and train-test (jackknife) evaluations and to record evaluation results to database. More information is included in the readme.txt file and the Javadoc files. The code can be used with a MySQL database. To use a different database software, other JDBC drivers might be required. [file 1471-2105-13-61-S5.gz › java_code/utils/doc/uk/ac/ed/inf/utils/database/TableRow.html]

TableRow


---


|  |  |  |
| --- | --- | --- |
| **Overview** \| **Package** \| **Class** \| **Use** \| **Tree** \| **Deprecated** \| **Index** \| **Help** | |  |
| **PREV CLASS**   **NEXT CLASS** | **FRAMES**    **NO FRAMES**     **All Classes** |  |
| SUMMARY: NESTED \| FIELD \| CONSTR \| METHOD | DETAIL: FIELD \| CONSTR \| METHOD |  |


---


## uk.ac.ed.inf.utils.database Class TableRow

```
java.lang.Object
  uk.ac.ed.inf.utils.database.DbManaged
      uk.ac.ed.inf.utils.database.TableRow
```

---

``` public class TableRow extends DbManaged ```

Contains the data for a generic row (a list of [table column, value] entries)
to be written to a database table.

**Version:**
:   9 Jun 2010

**Author:**
:   Luna De Ferrari luna.deferrari-at-ed.ac.uk

---

**Constructor Summary**

`TableRow(TableManager manager)`

`TableRow(TableManager manager,
java.util.TreeMap<java.lang.String,java.lang.String> columnToValueMap)`
  
          the auto-generated key of this row in its table (if available)

`TableRow(TableManager manager,
java.util.Vector<java.lang.String> columns,
java.util.Vector<java.lang.String> values)`


| **Method Summary** | |
| --- | --- |
| `boolean` | `checkRow()` |
| `boolean` | `checkRowFields()`             checks that the record values are paired to fields existing in the table |
| `boolean` | `checkRowValues()`             TODO Not implemented: check that each row value is of the appropriate datatype for the table field... |
| `java.lang.String` | `columnsToString()`             Returns a comma separated string of the values |
| `java.util.TreeMap<java.lang.String,java.lang.String>` | `getColumnAndValueMap()` |
| `java.util.Vector<java.lang.String>` | `getColumns()` |
| `TableManager` | `getTableManager()` |
| `java.lang.String` | `getValue(java.lang.String column)`             get the value for a certain column |
| `java.util.Vector<java.lang.String>` | `getValues()` |
| `boolean` | `isCorrect()` |
| `java.lang.String` | `put(java.lang.String key, java.lang.String value)`             Puts an entry in the table record map |
| `java.lang.String` | `toString()`             Returns a line with a comma-separated list of table column names followed by a line with the comma-separated list of values. |
| `java.lang.String` | `valuesToString()`             Returns a comma separated string of the values |

**Methods inherited from class uk.ac.ed.inf.utils.database.DbManaged**

`getDbConnection, getDbManager`

**Methods inherited from class java.lang.Object**

`equals, getClass, hashCode, notify, notifyAll, wait, wait, wait`

**Constructor Detail**

### TableRow

```
public TableRow(TableManager manager)
```

---


### TableRow

```
public TableRow(TableManager manager,
                java.util.TreeMap<java.lang.String,java.lang.String> columnToValueMap)
```

:   the auto-generated key of this row in its table (if available)

---


### TableRow

```
public TableRow(TableManager manager,
                java.util.Vector<java.lang.String> columns,
                java.util.Vector<java.lang.String> values)
```


**Method Detail**

### checkRow

```
public boolean checkRow()
```

---


### checkRowFields

```
public boolean checkRowFields()
```

:   checks that the record values are paired to fields existing in the table

    :   **Returns:**: true if the record values fields exist in the table

---


### checkRowValues

```
public boolean checkRowValues()
```

:   TODO Not implemented: check that each row value is of the appropriate
    datatype for the table field...

    :   **Returns:**: true if row values are of appropriate type for the corresponding field

---


### columnsToString

```
public java.lang.String columnsToString()
```

:   Returns a comma separated string of the values

    :   **Returns:**: a comma separated string of the values

---


### getColumnAndValueMap

```
public java.util.TreeMap<java.lang.String,java.lang.String> getColumnAndValueMap()
```

---


### getColumns

```
public java.util.Vector<java.lang.String> getColumns()
```

---


### getTableManager

```
public TableManager getTableManager()
```

---


### getValue

```
public java.lang.String getValue(java.lang.String column)
```

:   get the value for a certain column

---


### getValues

```
public java.util.Vector<java.lang.String> getValues()
```

---


### isCorrect

```
public boolean isCorrect()
```

---


### put

```
public java.lang.String put(java.lang.String key,
                            java.lang.String value)
```

:   Puts an entry in the table record map

    :   **Parameters:**: `key` -: `value` - **Returns:**: as in Map.put (returns the previous key value, or null if the key did not exist)

---


### toString

```
public java.lang.String toString()
```

:   Returns a line with a comma-separated list of table column names followed
    by a line with the comma-separated list of values.

    :   **Overrides:**: `toString` in class `java.lang.Object`

---


### valuesToString

```
public java.lang.String valuesToString()
```

:   Returns a comma separated string of the values

    :   **Returns:**: a comma separated string of the values


---


|  |  |  |
| --- | --- | --- |
| **Overview** \| **Package** \| **Class** \| **Use** \| **Tree** \| **Deprecated** \| **Index** \| **Help** | |  |
| **PREV CLASS**   **NEXT CLASS** | **FRAMES**    **NO FRAMES**     **All Classes** |  |
| SUMMARY: NESTED \| FIELD \| CONSTR \| METHOD | DETAIL: FIELD \| CONSTR \| METHOD |  |


---
